# Examining Hashtag Use of #blackboyjoy and #theblackmancan and Related Content on Instagram: Descriptive Content Analysis

**DOI:** 10.2196/34044

**Published:** 2022-08-01

**Authors:** Kofoworola D A Williams, Sharyn A Dougherty, Emily G Lattie, Jeanine P D Guidry, Kellie E Carlyle

**Affiliations:** 1 Department of Preventive Medicine Feinberg School of Medicine Northwestern University Chicago, IL United States; 2 Department of Health Behavior and Policy School of Medicine, Virginia Commonwealth University Richmond, VA United States; 3 Department of Medical Social Sciences Feinberg School of Medicine, Northwestern University Chicago, IL United States; 4 Robertson School of Media and Culture College of Humanities and Sciences, Virginia Commonwealth University Richmond, VA United States

**Keywords:** Black/African American men, mental health prevention, social media, Instagram, hashtags, content analysis, Black masculinity

## Abstract

**Background:**

Social media is widely accessible and increasingly utilized. Social media users develop hashtags and visual, text-based imagery to challenge misrepresentations, garner social support, and discuss a variety of mental health issues. Understanding how Black men are represented on social media and are using social media may be an avenue for promoting their engagement with and uptake of digital mental health interventions.

**Objective:**

The aim of this study was to conduct a content analysis of posts containing visual and text-based components related to representations of Black men’s race, gender, and behaviors.

**Methods:**

An exploratory, descriptive content analysis was conducted for 500 Instagram posts to examine characteristics, content, and public engagement of posts containing the hashtags #theblackmancan and #blackboyjoy. Posts were selected randomly and extracted from Instagram using a social network mining tool during Fall 2018 and Spring 2019. A codebook was developed, and all posts were analyzed by 2 independent coders. Analyses included frequency counts and descriptive analysis to determine content and characteristics of posts. Mann-Whitney U tests and Kruskal-Wallis H tests were conducted to assess engagement associated with posts via likes, comments, and video views.

**Results:**

Of the 500 posts extracted, most were image based (368/500, 73.6%), 272/500 (54.4%) were posted by an individual and 135/500 (27.0%) by a community organization, 269/500 (53.8%) were posted by individuals from Black populations, and 177/500 (35.4%) posts contained images of only males. Posts depicted images of Black men as fathers (100/500, 20.0%), Black men being celebrated (101/500, 20.2%), and Black men expressing joy (217/500, 43.4%). Posts (127/500, 25.4%) also depicted Black men in relation to gender atypical behavior, such as caring for children or styling their children’s hair. Variables related to education and restrictive affection did not show up often in posts. Engagement via likes (median 1671, *P*<.001), comments (*P*<.001), and views (*P*<.001) for posts containing #theblackmancan was significantly higher compared with posts containing #blackboyjoy (median 140). Posts containing elements of celebrating Black men (*P*=.02) and gender atypical behavior (*P*<.001) also had significantly higher engagement.

**Conclusions:**

This is one of the first studies to look at hashtag use of #blackboyjoy and #theblackmancan. Posts containing #blackboyjoy and #theblackmancan promoted positive user-generated visual and text-based content on Instagram and promoted positive interactions among Black and diverse communities. With the popularity of social media and hashtag use increasing, researchers and future interventional research should investigate the potential for such imagery to serve as culturally relevant design components for digital mental health prevention efforts geared towards Black men and the communities they exist and engage with.

## Introduction

### Background

Social media is extremely popular and widely accessible [1,2]. More than 80% of young adults use social media platforms extensively [1], with typical activity exceeding 6 hours per week [2,3]. Social media networking sites are also now dominant sources of health information and health promotion [4,5]. According to the Pew Research Center, 72% of adult users search for news and health information online [6,7] and 26% of adults in the United States learn about health experiences from others on social media platforms [6]. Despite the increasing popularity of social media and its use, user statistics about Black men on these platforms is extremely limited. However, more than 65% of Black adults in the United States are using social media platforms on a daily basis [1]. Understanding how Black men are represented on and use social media may be an avenue for promoting their engagement with and uptake of digital mental health interventions [8]. The conversations on social media include discussions about sensitive mental health topics and the sharing of individuals’ personal experiences with mental health issues [9], as well as interactions where people are connecting with one another and seeking social support [8]. Content analyses on photo-based platforms, such as Instagram, could provide a unique approach for researchers to examine how social media platforms can promote mental well-being among underserved groups.

### Instagram and #theblackmancan #blackboyjoy

Instagram, launched in 2010, is a free, widely accessible, photo- and video-sharing social networking site [10]. In the United States, Instagram is one of the more popular social networking platforms, more widely used than Twitter [11], with a national user base of over 170 million and a global user base of over 1.3 billion people [12,13]. In particular, Instagram is very popular among young Black adults [14,15].

The popularity of hashtags, which are phrases or words strung together with a hashtag (#) placed in front of it, has grown exponentially and gained traction on platforms such as Instagram. Hashtags are widely used and can be started by anyone, defying the limits typically associated with traditional media and news outlets, and can lead to significant political and social movements [15,16]. In 2017, the hashtag #MeToo sparked a social movement in global support of sexual assault and harassment survivors. Within the Black community, the hashtag #BlackLivesMatter, coined in 2013, continues to be an important campaign, set against violence and systemic racism toward Black people. Similarly, celebrities, such as Kid Cudi, and athletes, such as Dak Prescott, used social media and relevant hashtags to discuss their personal issues with bipolar disorder and depression. The practical effects of these hashtags and movements are overwhelming, resulting in structural changes in both the entertainment industry and society at large. Despite this increased popularity and galvanizing nature of hashtags, there is little research focusing on the use of hashtags among diverse communities and the potential role they can play in mental health promotion.

The hashtags #theblackmancan and #blackboyjoy are popular among the Black male and Black community. The hashtag #theblackmancan was created by a nonprofit organization, TheBlackManCan, Inc., founded by Brandon Frame, to share stories aimed at empowering Black boys and men [17]. TheBlackManCan, Inc. started as a blog in April 2010, with the aim to empower Black men and has since become a “social community that reflects positive images of Black boys and men of color” [17]. The organization’s Instagram now has over 898,000 followers [18]. The #blackboyjoy is another hashtag created specifically to promote positive imagery and images about Black boys and men to counter the negative stereotypes that are placed on Black men in today’s society [19,20]. This hashtag was popularized in 2016 by Danielle Young [20], writer for *The Root*, an African American–oriented online magazine, after watching Black male musician and producer Chance the Rapper displaying happiness at an award show. This hashtag, used over 1,832,953 times on Instagram [21], has evolved, and is widely used to create and highlight online spaces for Black men to express and display sentiments of joy, happiness, and laughter. To date, there are no published analyses looking at these hashtags. Importantly, there is little research examining social media hashtag use as a possible avenue for mental health promotion, creating a gap in our understanding of the efficacy of utilizing these, and other hashtags, in promoting culturally relevant design elements for interventional efforts geared toward minority men’s mental health.

### Related Work

Much of our knowledge of social media platforms and related user-generated content excludes social representations of Black men [22], focuses heavily on understanding data dissemination [23], and is often focused on Twitter [24,25]. For example, one study examined characteristics of #fitspiration content on multiple social media platforms, finding that the majority of the posts depicted women, and presented imagery of women reinforcing the “thin” ideal, which is negatively associated with unhealthy behaviors [26]. This analysis, among others [27], provides direction for researchers investigating which ideals are most pervasive in reinforcing unhealthy social norms. Researchers, then, can develop messages that appropriately counter such norms and, instead, encourage healthy behaviors [26]. Few studies have taken this approach in relation to Black men’s health behaviors and mental health, making this a critical focus area. Content analyses have also examined the formation of social representations via images, which are important in health-promotion efforts [22]; examining health behaviors; understanding risk for health issues among underserved populations; and learning about the lived experiences of marginalized individuals [28]. However, there remains a paucity of content analyses focused on Black men, their interests, and their lived experiences. This lack of understanding and knowledge likely reinforces stigmatized beliefs related to Black men, their mental health, and their overall lived experiences.

### Guiding Framework

There is a growing literature base in social media content analyses, providing insight into the role post content and hashtags can play in understanding the lived experiences of traditionally excluded populations. Such insight creates opportunities for researchers to design mental health–promotion efforts that are culturally and contextually relevant to those they wish to serve. Because there were no existing codebooks on the topic, this study utilized a framework informed by formative literature and relevant theoretical concepts, including Tyree et al [29] and Gray’s [30] framework for Black masculinity, the Gender Role Conflict Scale, and aspects from The Black Man Can Institute’s mission statement and Black Boy Joy’s website.

### Masculinity and Black Masculinity

Ideals associated with masculine norms are important to assess as social constructions of masculinity are significant barriers to the uptake of mental health–promotion efforts and often negatively impact mental well-being among Black men [31-33]. According to gender role conflict constructs [34], due to masculine norms and expectations, men have difficulty balancing workplace and family demands and are socialized to be competitive, emotionally restrictive, and refrain from showing affection for other men [34]. The extent to which men adhere and conform to these ideals can cause high levels of stress for men, as well as for their interpersonal relationships [35]. Studies report direct links between measures of masculine adherence and lack of adherence to medical and mental health–related help and treatment [35]. Black men are not only socially expected to conform to masculine norms but are also presented with unique societal expectations, resulting from negative stereotypes associated with Black masculinity and Black masculine positionality [29,30]. These stereotypes often stem from misrepresentations in the media [36]. Jackson [37] and Tyree and colleagues [29] provide a framework of Black masculinity and positionality, proposing that there needs to be a redefining in which Black men are defined by ideals more closely associated with their experiences as Black men in society [29,30]. Black men often relate to certain ideals of masculinity—struggle, recognition, independence, achievement, and community—that define their manhood, masculinity, and position in society [29,30]. Other characteristics of Black men that are positive and ascribed to include presenting a “cool pose” or exhibiting a comedic nature [29,30]. This study aims to analyze posts related to Black men’s masculine identity. Findings may be translated to other work, serving as targets for conducting mental health work within the realm of social media.

### Current Study

This exploratory, descriptive study employed content analysis to identify characteristics of Instagram post content containing the hashtags #blackboyjoy and #theblackmancan. This content analysis examines how diverse users, including Black men, are generating content and social media messages that present positive images and lived experiences of Black men. We also aimed to examine public engagement with posts to see how visual and text-based social media messages and user-generated content promoted engagement or interactions. The overarching research questions were:

- How are posts containing #blackboyjoy and #theblackmancan portrayed on Instagram and what are the characteristics of these posts?
- To what extent do these posts challenge stereotypes associated with Black men’s race and gender?
- Do these posts garner interactions or support?

## Methods

### Study Design

A social media content analysis [38,39] was conducted to examine the content of posts related to Black men on a social media platform, Instagram. A random sample of public Instagram posts was collected using Netlytic [40], a web-based social network mining tool, during the Fall of 2018 and Spring of 2019. Posts containing the hashtags of interest, #theblackmancan and #blackboyjoy, were extracted. Posts were assessed visually for inclusion in analysis if they (1) contained either or both hashtags and (2) were in English or could be translated into English using Instagram’s translation feature. Posts were excluded if they did not contain either hashtag or if the link for access was no longer active at the time of coding. The poster source, caption, post, and post components were examined for this study. Posts were coded for a variety of variables related to the study’s aims and objectives and were coded by 2 independent coders (KDAW and SAD). Two coders received extensive training on the utilization of the codebook. An iterative process was conducted in which weekly meetings were established to discuss discrepancies, determine consensus, and reach agreement between coders; 10.0% (n=50) of the Instagram posts were independently analyzed by the coders to reach an agreement as assessed by Cohen κ [41]. Guidelines for reliability analyses in content analyses suggest that there must be agreement on at least 10% of the study’s sample with a κ statistic near the recommended cut-off value of 0.70 [41]. For this study, intercoder reliability for coding and analysis showed a coefficient of agreement (κ) between coders no lower than 0.69.

### Codebook Development

Given the dearth of evidence on content analyses related to Black men and relevant hashtags, there is no established codebook to use or adapt for this analysis; therefore, a codebook was developed specifically for this content analysis (codebook is available in Multimedia Appendix 1). The first author (KDAW) first developed and operationalized codes based on current literature, study objectives, and variables of interest. The codebook went through multiple phases of revisions based on feedback from 2 university faculties with expertise in conducting quantitative content analyses on social media. These revisions focused mostly on the development and iteration of codes related to visual components of the posts. After revisions were made, the codebook was reviewed by 2 Black, male graduate students. These men’s perspectives in choosing and developing codes were important to ensure appropriate interpretation of Instagram posts including variables related to Black culture and Black men. This technique mirrors member checking, providing feedback on study methods, and approaches by members of the intended target population, which promoted validity of the research methods and findings [42].

### Variables of Interest

#### Post Characteristics

The initial set of codes focused on visual and face-value content and included codes related to the visual components. Variables included focused on poster source (ie, who is posting), the kind of visual (eg, text or image based), and people present in post (eg, number of people), among other aspects.

#### Post Content

Variables were then added to assess context and content. Such variables assessed aspects related to masculinity and gender roles and were informed by items from the Gender Role Conflict Scale, Gray [30] and Tyree et al’s [29] framework for Black masculinity, as well as components of The Black Man Can Institute’s mission statement [17] and Black Boy Joy’s website. For example, posts were coded for whether (1=Yes) or not (0=No) they indicated aspects related to a creation of a safe space, displays of community service, the celebration of Black boys and men, protection, and if men could be seen displaying joy (ie, a boy or man smiling). Posts were also coded for whether (1=Yes) or not (0=No) they focused on aspects related to Black boys or men in educational contexts, receiving or giving mentorship, and men’s attire.

#### Post Engagement

Codebook variables also included codes for assessing public engagement. The included variables were informed by engagement features typically used by Instagram users, analyzing how many likes, comments, and video views (all Instagram application features) were attached to each post.

### Data Analysis

All statistical analyses were conducted using SPSS, version 26 (IBM, Inc.). Upon completion of data collection and coding, frequency counts and descriptive statistical analysis of the coded variables were conducted. Three different types of engagement, namely, likes, comments, and views for videos, were evaluated using nonparametric tests, as Instagram engagement frequencies were not normally distributed. Specifically, Mann-Whitney *U* tests were used to investigate differences in the level of public engagement for variables containing a range of dichotomous variables; and Kruskal-Wallis *H* tests were used to investigate differences in engagement for nominal variables with 3 or more levels.

### Ethical Considerations

This study did not directly involve human subjects and did not meet the regulatory definition of human subjects’ research; therefore, this study was excluded from institutional review board review at the institution in which this study took place.

## Results

### Characteristics of Posts

The first research question focused on how posts containing #blackboyjoy and #theblackmancan are being portrayed on Instagram and the characteristics of these posts (see Table 1 for descriptive analyses). Approximately half of the sample either included the hashtag #theblackmancan (248/500, 49.6%) or #blackboyjoy (225/500, 45.0%). Only 27/500 (5.4%) of the posts included both hashtags. All posts contained a poster source, that is, the person or entity who posted the actual post with the hashtag. Of 500 posts, 272 (54.4%) were posted by an individual person, whereas 135 (27.0%) were posted by a community organization. It is important to note that the community organization code captured posts that were also posted by The Black Man Can Institute; however, the software that extracted the posts did so randomly and only extracted posts based on the hashtag itself.

Among posts that had a poster source, only 17/500 (3.4%) were posted by commercial sites. As many as 194/500 (38.8%) of the posts were posted by men and 81/500 (16.2%) were posted by women. Of these posters, 269/500 (53.8%) were posted by individuals from the Black populations. The types of visuals (ie, image or video) included were still images (368/500, 73.6%) and videos (106/500, 21.2%). Among these visuals, 177/500 (35.4%) contained only males, and 179/500 (35.8%) posts contained individuals who were Black. Of the 500 posts, 272 (54.4%) posts containing multiple people were Black. Furthermore, of these posts containing multiple people, 184/500 (36.8%) were of mixed gender and 122/500 (24.4%) posts contained multiple males.

**Table 1:** Characteristics, descriptions, and post content among posts tagged with #theblackmancan and #blackboyjoy (N=500).

Variable and category/item			Value, n (%)
**Characteristics**			
	**Hashtag**		
		#blackboyjoy	248 (49.6)
		#theblackmancan	225 (45.0)
		Both	27 (5.4)
	**Poster source**		
		Individual	272 (54.4)
		Commercial	17 (3.4)
		Community Organization	135 (27.0)
		Poster source gender: male	194 (38.8)
		Poster source race: Black	269 (53.8)
	**Visual type**		
		Image	368 (73.6)
		Text	7 (1.4)
		Mix of image/text	12 (2.4)
		Drawing	4 (0.8)
		Video	106 (21.2)
	**Person in visual**		
		Only one person present	179 (35.8)
		Individual present is Black	179 (35.8)
		Individual present is male	177 (35.4)
	**Multiple persons in visual**		
		Only multiple people present	312 (62.4)
		Single race: Black	272 (54.4)
		Single gender: only men	122 (24.4)
	**Multiple persons in visual can be^a^**		
		Children	32 (6.4)
		Friends	18 (3.6)
		Colleagues	15 (3.0)
		Love	48 (9.6)
		Family	55 (11.0)
		Brotherhood	17 (3.4)
		Fatherhood	100 (20.0)
		Other	35 (7.0)
**Content focused**			
	**Related to #blackboyjoy**		
		Safe space	3 (0.6)
		Community service	4 (0.8)
		Celebration of boys	101 (20.2)
		Protection	19 (3.8)
	**Related to #theblackmancan**		
		Finding joy	217 (43.4)
		Education	44 (8.8)
		Mentorship	9 (1.8)
		Casual attire wear	254 (50.8)
		Business casual wear	36 (7.2)
		Professional attire wear	36 (7.2)
		Formal attire wear	50 (10.0)
		Stereotypically dressed	6 (1.2)
	**Related to gender roles and conflict**		
		Gender behavior	127 (25.4)
		Restricted affection	29 (5.8)
		Restricted emotion	7 (1.4)
		Conflict	5 (1.0)
		Power and competition	17 (3.4)
		Graduation themed	9 (1.8)
		Cool pose	154 (30.8)

^a^These variables were visually assessed along with the captions associated with posts.

### Post Content

**Figure 1:**
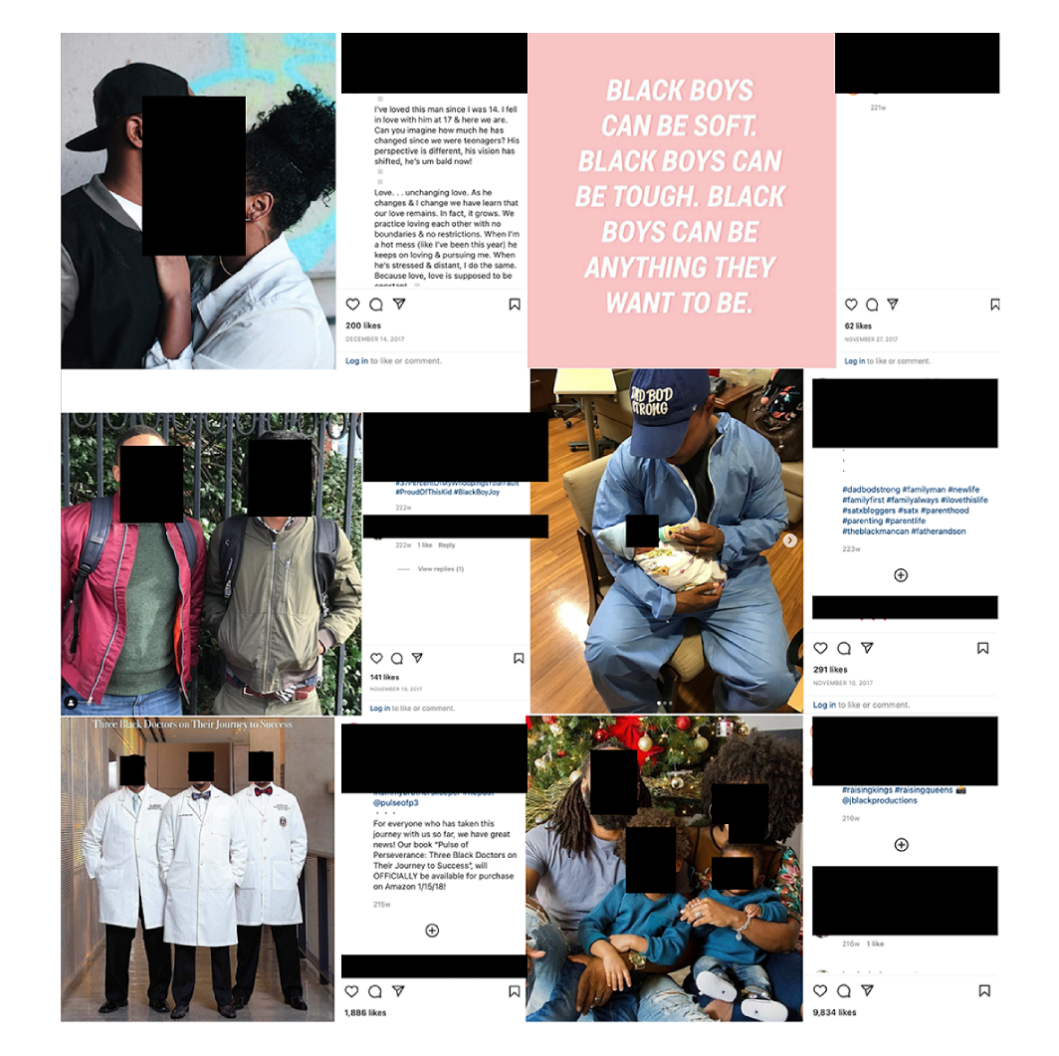
Examples of posts portraying aspects related to finding joy, fatherhood, and celebration of Black boys or men.

The second research question focused on how these posts challenge stereotypes associated with Black men’s race or gender and to what extent the public engages with these posts. For the variable assessing the type of imagery depicted in the visual (Table 1), descriptive analyses show that 32/500 (6.4%) posts solely depicted children, 48/500 (9.6%) posts portrayed praise for significant others, and 55/500 (11.0%) posts showed portraits of families (father, mother, and a child or children); 100/500 (20.0%) posts presented images or videos of Black men portrayed as fathers (see Figure 1 for examples of posts).

Among the codes assessing values associated with the mission statement of The Black Man Can Institute, analyses revealed posts celebrating or showing praise for Black men/boys (101/500, 20.2%) and Black men/boys smiling and expressing joy (217/500, 43.4%). Attire of individuals in these posts were also of interest, as negative stereotypes often show Black men dressed in a manner that is indicative of being criminals and thugs. As many as 254/500 (50.8%) posts included persons who were dressed casually. Other posts showed persons who were dressed formally (50/500, 10.0%), business casually (36/500, 7.2%), and professionally (36/500, 7.2%); 1.2% (6/500) of the posts showed Black men dressed negatively or similar to that of a gang member or thug. Specific to gender roles, posts (127/500, 25.4%) showed men engaging in gender atypical behavior, such as caring for children, doing a child’s hair, or engaging in a form of dance other than hip-hop (eg, ballet). However, not necessarily surprising, only 29/500 (5.8%) posts showed men who challenged the norm of restricted affection toward or among men.

### User Engagement

Analyses revealed statistically significant differences, showing that engagement by likes was higher for posts using #theblackmancan (median 1671) compared with engagement scores of posts only using #blackboyjoy (median 140, *P*<.001). Similarly, for comments, there were significantly higher levels of engagement for posts using #theblackmancan compared with those using #blackboyjoy (*P*<.001). For engagement via views, there were significant differences in engagement between posts using #blackboyjoy and posts using both (*P*<.001) such that #theblackmancan showed higher levels. Mann-Whitney *U* tests were conducted for the variables in Table 2. For celebration of boys, there were significant levels of engagement across all types of engagement (likes, *P*=.02; comments, *P*<.001; views, *P*<.001). Similarly, for posts including men engaging in gender atypical behaviors (eg, taking care of children, braiding child’s hair), there were higher levels of engagement displayed by likes (*P*<.001), comments (*P*<.001), and views (*P*=.001). Engagement by likes, comments, and views varied for several variables examined, and there were no significant differences for any types of engagement among multiple variables. Specifically, there were no significant differences in types of engagement for posts containing the mentorship (likes, *P*=.83; comments, *P*=.47; views, *P*=.39), community service (likes, *P*=.95; comments, *P*=.64; views, *P*=.30), safe space (likes, *P*=.13; comments, *P*=.16; views, *P*=.71), education (likes, *P*=.57; comments, *P*=.47; views, *P*=.65), conflicts between work and family (likes, *P*=.89; comments, *P*=.28; views, *P*=.87), or competition/power variables (likes, *P*=.05; comments, *P*=.22; views, *P*=.22).

**Table 2:** Median engagement scores for posts containing race and gender variables in this analysis (N=500 unless otherwise noted).

Variable and engagement variable			Median (present)		Median (absent)		*U* test		Z score		*P* value
**Celebration of boys**											
	Likes	1076.00		370.00		24,115.500		3.058		.002^a^	
	Comments	27.00		15.00		24,494.000		3.351		<.001^a^	
	Views	0		0		23,588.000		3.696		<.001^a^	
**Finding joy**											
	Likes	619.00		410.00		32,658.500		1.220		.22	
	Comments	17.00		17.00		31,666.000		0.600		.55	
	Views	0		0		34,830.000		3.591		<.001^a^	
**Atypical gender behavior**											
	Likes	1779.00		313.00		31,077.000		5.256		<.001^a^	
	Comments	43		13		28,941.500		3.739		<.001^a^	
	Views	0		0		26,968.000		3.254		.001^b^	
**Restricted affection**											
	Likes	231.00		491.00		6651.000		–0.236		.81	
	Comments	18.00		17.00		6519.500		–0.411		.68	
	Views	0		0		8478.500		3.045		.002^b^	
**Protection**											
	Likes	3449.00		424.00		6076.500		2.440		.02^a^	
	Comments	67.00		17.00		6055.500		2.406		.02^a^	
	Views	0		0		4826.000		0.579		.56	
**Mentorship**											
	Likes	403.00		462.00		2304.500		0.221		.83	
	Comments	33.00		17.00		2519.500		0.722		.47	
	Views	0		0		1944.000		–0.862		.39	
**Community service**											
	Likes	279.00		475.50		972.000		–0.069		.95	
	Comments	14.00		17.00		1125.500		0.464		.64	
	Views	0		0		778.000		–1.037		.30	
**Safe space**											
	Likes	102.00		489.00		364.500		–1.527		.13	
	Comments	7.00		17.00		394.000		–1.409		.16	
	Views	0		0		813.000		0.377		.71	
**Education**											
	Likes	455.00		458.00		10,551.000		0.567		.57	
	Comments	23.00		17.00		10,688.000		0.717		.47	
	Views	0		0		10,333.000		0.459		.65	
**Wedding party (attire; n=63)**											
	Likes	2854.50		941.00		491.500		1.316		.19	
	Comments	37.00		36.00		426.500		0.327		.74	
	Views	0		0		448.000		1.028		.30	
**Atypical gender behavior**											
	Likes	1779.00		313.00		31,077.000		5.256		<.001^a^	
	Comments	43.00		13.00		28,941.500		3.739		<.001^a^	
	Views	0		0		26,968.000		3.254		.001^a^	
**Restrictive affection**											
	Likes	231.00		491.00		6651.000		–0.236		.81	
	Comments	18.00		17.00		6519.500		–0.411		.68	
	Views	0		0		8478.500		3.045		.002^a^	
**Restrictive emotion**											
	Likes	879.00		452.00		1721.500		–0.011		.99	
	Comments	15.00		17.00		1647.000		–0.207		.84	
	Views	5237.00		0		2817.000		4.009		<.001^a^	
**Conflicts between family and work**											
	Likes	373.00		489.00		1284.000		0.145		.89	
	Comments	67.00		17.00		1582.000		1.072		.28	
	Views	0		0		1199.000		–0.167		.87	
**Competition/power**											
	Likes	122.00		519.00		2968.500		–1.942		.05	
	Comments	6.00		18.00		3390.500		–1.222		.22	
	Views	0		0		4629.000		1.223		.22	
**Cool pose**											
	Likes	352.50		607.50		24,622.500		–1.354		.18	
	Comments	18.00		17.00		26,060.000		–0.390		.70	
	Views	0		0		23,774.500		–2.681		.007^b^	
**Comedic presence**											
	Likes	2332.00		426.00		4614.000		1.772		.08	
	Comments	106.00		16.00		5420.500		3.236		.001^a^	
	Views	7575.00		0		5817.000		5.514		<.001^a^	

^a^Significant at *P*<.05.

^b^Significant at *P*<.05, but not significant in practice as both medians are 0.

Kruskal-Wallis *H* tests showed significant differences in engagement via comments (χ^2^_7_=15.961, *P*=.002) for the attire type variable and via views (χ^2^_9_=19.490, *P*=.02) for the group type variable. There were differences in engagement scores for the group type variable and attire type variable. The group type variable included categories such as children, friends, colleagues, family, brotherhood (see the “Multiple persons in visual can be” variable in Table 1). According to analyses, there were significant differences between posts containing the family variable (median 0) and those containing the “other” variable (median 54.00, *P=*.006). The attire type variable included categories such as an individual wearing casual clothes, business casual, professional, formal, or stereotypically dressed clothing (ie, thug garb; Table 1). There were significant differences between posts containing the casual (median 0) and other variable (median 29.00, *P*=.03) and between the casual variable and formal variable (median 43.50, *P*=.05), but not among other groups.

## Discussion

### Principal Findings

This study analyzed Instagram posts containing the hashtags #blackboyjoy and #theblackmancan. The findings offer direction for researchers who wish to use visual and text-based content in mental health–promotion efforts geared toward Black men.

Overall, most posts were primarily image based with only a small number including videos or video-based images. Analyses showed that there were few posts that portrayed depictions related to education and restricted affection, and posts depicting ideals particular to #blackboyjoy included imagery where men were shown celebrating Black boys and men and finding joy. When analyzing whether posts were posted by an actual person, group, or community organization, we see that Black men and communities are, in fact, using these hashtags. Community organizations, in particular, used the #theblackmancan hashtag more often compared with #blackboyjoy. We also see that the images analyzed in this study foster positive interactions from viewers and users on Instagram.

According to analyses of engagement, public engagement was positive for posts containing variables related to celebrating men and boys, men engaging in gender atypical behavior, and posts displaying sentiments of protection. In addition, engagement via viewership was high for the following variables: cool pose, finding joy, restrictive affection and restrictive emotion, and comedic presence. The #theblackmancan hashtag showed more public engagement, eliciting more likes and comments from the public, suggesting that ideals and expressions related to the #theblackmancan were more popular and relatable at the time of analysis and produced more aesthetically appealing content than #blackboyjoy. It is difficult to ascertain why this is the case; however, it may be due to social media users’ access or the fact that this hashtag is influenced by the organizational reach of The Black Man Can Institute. It could be that the #blackboyjoy movement, though popular, did not have the same reach. However, this is unclear and difficult to establish for certain as the #blackboyjoy movement received a lot of national attention by a large group of people, including celebrities who have either endorsed or not endorsed the hashtag’s use.

### Comparison With Prior Work

The finding that most posts were image based was expected as Instagram is primarily a photo-sharing platform and indicates that, despite the social media having a feature for the posting of videos, the posting of still images and pictures remain the primary behavior on this social media platform [43]. Consistent with emerging research, many of the images expressed personal beliefs and values, which has relevance for recent research examining the impact of photo sharing and photo elicitation and its role in mental health prevention and promotion [44]. In community-based participatory work, researchers utilize similar methods to understand the experiences of marginalized populations in an effort to promote health and understand lived experiences of minority populations [45].

Notably in this study are posts including images of mothers or wives offering support for their significant others and showing appreciation for the Black men and boys in their lives. Although these images showed up in less than 50% (101/500, 20.2) of the posts, this representation of “celebrating Black men” is important and suggests how community, and even researchers, can utilize outlets such as Instagram to empower Black men and men of color and their accomplishments, which is atypical according to traditional images put forth by traditional media [46]. Images with Black men being celebrated also included sentiments of men with their families. The posts in this study depicted Black men as family men who engaged in “gender atypical behavior” (127/500, 25.4%) such as men taking their children to work or doing their daughter’s hair. This positive representation of Black men engaging in fatherhood is often underrepresented or inaccurately represented in the media, such that men are portrayed as absent in the household or intentionally disengaging from family responsibilities [47,48], leading to reinforcement of negative stereotypes [49] that inevitably impact Black men [50,51].

Increased depictions of Black men in a positive light will promote positive representation of Black men within digital mental health spaces that are visual based. This representation can then lead to men’s increased interest, engagement, and acceptability of current and future mental health visual-based interventions [22]. This study’s findings, related to Black men displaying joy and positive engagement, highlight a potential visual or text-based characteristic that can be incorporated into a social media, mental health message geared toward Black men. There is already work being done in the mental health message space such that Robinson and colleagues [52] found that social media–based, suicide prevention messages are acceptable and safe among young adults. A recent paper by Seidler and colleagues [53] set forth a call for action for developing “gender-sensitive” and appropriate mental health care and how such approaches should emphasize a redefining of men’s masculinities. This redefining can include positive imagery as that seen in this study and will aid in our ability to increase representation of Black males and their engagement with mental health prevention efforts, specifically help-seeking and mental health prevention campaigns [53].

There were few posts that included depictions of men related to education and restricted affection. This finding can be related to the fact that misrepresentations and stereotypes associated with Black men being uneducated [54] and Black men being unable to show affection are not as easy to counter in this medium. These ideals, combined with social norms criminalizing Black men and stigmatizing mental health within this community, (1) reinforce unrealistic expectations related to unhealthy masculine ideals, such as restrictive emotion [31]; and (2) make it particularly challenging and uncomfortable for Black men to engage with health-related efforts and programming [28]. Few depictions of men related to education may also be reflective of educational disparities that continue to exist for Black men as there are higher dropout rates and lower retention among this group [55]. Such disparities and representations may be harder to challenge in this medium; however, future research would be appropriate to investigate this further.

### Future Work

With social media constantly evolving, further research should be conducted to examine how prevalent hashtags can counter and reshape narratives that promote ideal health behaviors and mental health outcomes among Black men [56]. There are various approaches and avenues that researchers can utilize as we move forward in the field of digital mental health. One approach is further examination of user-generated, asset-based content that gives insight into the various images and imagery Black men choose to express themselves on a digital-based platform and using digital tools most relevant to them, such as hashtag use. From this study, we see that Black men are portraying themselves in a different light but also show which factors or norms may be more pervasive and relevant to Black men and their communities. This “flipping of the narrative” can hold much promise for the future of mental health prevention geared toward minority men and other populations such that programming can take a visual-based approach, utilizing positive imagery that is relevant to minority populations.

Researchers can further examine study variables as potential mutable factors for intervention work and include intervention design elements that are culturally appropriate, contextually relevant to Black men and, eventually, once translated and adapted, other minority men and their communities. For example, in the posts, there were a high number of images containing visuals of Black men being celebrated. Figuring out how to incorporate this positive aspect into programming and visual-based campaigns will draw Black men in to participate in research as well as empower them, increasing their self-efficacy and promoting adherence of therapeutic treatments in which the campaigns are advertising.

Findings from this study also begin to highlight how a social media platform, such as Instagram, can be manipulated to promote sentiments, such as celebrating men or destigmatizing restricted affection and emotion. Researchers, clinicians, and mental health professionals can expand on the study findings, engaging in intervention work that focuses on Instagram and other relevant platforms, and consider how this generated content promotes health and mental health support messages. For example, within this study, there is some indication of the potential role of peer support via family and loved ones, who are using these hashtags and posting appropriate and relatable content. Future research should investigate how to combine the use of visual-based and text-based images with human support to create and implement future mental health and suicide prevention efforts [9] that are useful and sustainable within Black male communities. Similar to photo elicitation work [57], further exploration into image-based messaging will allow researchers to examine and begin to understand how images promote ideals related to mental health, their lived experiences, and even, their mental and emotional safety [28,44,57]. In addition, as we see community organizations may be engaging with this hashtag, and others like it, it would be important to conduct work with and partner with community organizations to foster men’s engagement on a broader level and with wider impact.

### Limitations

As with any study, there are limitations to acknowledge. Although efforts were made to address the concern for human error in this study, there may have been posts falsely excluded due to misjudgment by coders. There are variables included in this analysis that only allowed for “other” or “unsure” or “uncertain” or “cannot tell” and, due to the subjective nature of content analyses, this may have led coders to miss variables, limit codes, or include irrelevant variables. There may have been posts unable to be included in the analysis because an Instagram user’s profile was private or the link to a profile at time of analysis was deemed “no longer existing.” Mentions (an Instagram feature where the poster uses an “@” symbol followed by an individual’s Instagram handle) were not coded in this study. Coders, then, did not code for this under the category Public Engagement. At the time of analysis, the hashtags, #blackboyjoy and #theblackmancan, though still used, were popular and used often; however, in posts, these hashtags were not the only ones used. Our analyses only aimed to extract posts that contained these hashtags and posts included in this analysis could contain other hashtags. Despite these limitations, the codebook was thoroughly developed and updated continuously for a period of over 5 months in collaboration with members who matched the study’s sample by gender and race to ensure there were codes appropriate in capturing variables associated with study goals and objectives.

### Conclusions

Digital mental health interventions can reduce health disparities and improve access to service; however, there is a paucity of digital mental health work geared toward Black men. When members from underserved and underrepresented populations see themselves in prevention efforts, they are more likely to engage [22]. By examining social media content celebrating Black men, this study can inform the design of digital tools that are visual based and relevant. Developing technology and digital programming effective in promoting mental health among minority populations and improving related health behaviors are of vital importance. This study is one of the first to explore the use of the hashtags #blackboyjoy and #theblackmancan on Instagram, illustrating the potential for hashtags, as digital tools, that express Black men’s values and beliefs, allowing these men to illustrate their lived experiences as Black men in society. As social media users continue to access the platforms to share experiences and communicate, there is a need to further examine how members from underserved populations interact with social media and how this technology can be harnessed to promote their well-being.

## Appendix

Multimedia Appendix 1 Codebook for: Examining social media and hashtags as digital tools: A descriptive content analysis of #blackboyjoy and #theblackmancan on Instagram.
