# Supplementary material for: Examining Hashtag Use of #blackboyjoy and #theblackmancan and Related Content on Instagram: Descriptive Content Analysis
Source: JMIR Form Res. 2022 Aug 1;6(8):e34044. doi: 10.2196/34044 (PMC9379788; doi:10.2196/34044)
Supplement: Multimedia Appendix 1 [file formative_v6i8e34044_app1.docx]

| **START OF SHEET** | | | |
| --- | --- | --- | --- |
|  | | | |
| **FIRST SECTION** | | | |
| ID/Post # |  | | |
| Keyword |  | | |
| Link |  | | |
| HASTAG Contains: | | | |
| BlackBoyJoy | 1 |  |  |
| TheBlackManCan | 2 |  |  |
| Both | 3 |  |  |
| **POSTER SOURCE** | | | |
| Individual | 1 |  |  |
| Commercial | 2 |  |  |
| Community Org | 3 |  |  |
| Public Health/Health-oriented | 4 |  |  |
| Academic | 5 |  |  |
| Other | 6 |  |  |
| Cannot Tell | 99 |  |  |
| If an Individual: | | | |
|  | Male | 1 |  |
|  | Female | 2 |  |
|  | N/A | 3 |  |
|  | Cannot tell | 99 |  |
| If an individual: | | | |
|  | Black | 1 |  |
|  | White | 2 |  |
|  | Latino | 3 |  |
|  | Asian | 4 |  |
|  | Native American | 5 |  |
|  | Other | 6 |  |
|  | N/A | 7 |  |
|  | Cannot tell | 99 |  |

| **VISUAL TYPE** | | | |
| --- | --- | --- | --- |
| Primarily Image | 1 |  |  |
| Primarily Text | 2 |  |  |
| Mix of text and image | 3 |  |  |
| Infographic | 4 |  |  |
| Drawing | 5 |  |  |
| Video | 6 |  |  |
| Other | 7 |  |  |
| Visual Contains and Individual? | | | |
|  | Yes | 1 |  |
|  | No | 0 |  |
| If an Individual: | | | |
|  | Male | 1 |  |
|  | Female | 2 |  |
|  | N/A | 3 |  |
|  | Cannot tell | 99 |  |
| If an individual: | | | |
|  | Black | 1 |  |
|  | White | 2 |  |
|  | Latino | 3 |  |
|  | Asian | 4 |  |
|  | Native American | 5 |  |
|  | Other | 6 |  |
|  | N/A | 7 |  |
|  | Cannot tell | 99 |  |
| Visual Contains Multiple People? | | | |
|  | Yes | 1 |  |
|  | No | 0 |  |
| If multiple people: | | | |
|  | Mixed Race |  |  |
|  | Single race |  |  |
|  | N/A |  |  |
|  | Cannot tell |  |  |
| If Single Race: | | | |
|  | Black | 1 |  |
|  | White | 2 |  |
|  | Latino | 3 |  |
|  | Asian | 4 |  |
|  | Native American | 5 |  |
|  | Other | 6 |  |
|  | N/A | 7 |  |
|  | Cannot tell | 99 |  |
|  |  |  |  |
| If multiple People | | | |
|  | Mixed Group | 1 |  |
|  | Single Group Female | 2 |  |
|  | Single Group Male | 3 |  |
|  | N/A | 4 |  |
|  | Cannot tell | 99 |  |
|  |  |  |  |
| If multiple people, what can be seen in visual? | | | |
|  | Children | 1 | E.g. If someone looks younger than a teenager |
|  | Friends | 2 |  |
|  | Colleagues | 3 |  |
|  | Significant Other/Love | 4 |  |
|  | Family | 5 |  |
|  | Brotherhood/Frat | 6 |  |
|  | Fatherhood | 7 |  |
|  | Other | 8 |  |
|  | N/A | 9 |  |
|  | Cannot tell | 99 |  |
| Visual contains Quote: | | | |
|  | Yes | 1 |  |
|  | No | 0 |  |
| Is quote inspirational/motivational? | | | |
|  | Yes | 1 |  |
|  | No | 0 |  |
|  | N/A | 2 |  |
| Where does visual seem to take place? | | | |
|  | Home (i.e. inside, directly outside, driveway) | 1 |  |
|  | School or classroom | 2 |  |
|  | Public Venue | 3 |  |
|  | Street (e.g. residential area, corner store, outside a gym, parking lot, etc.) | 4 |  |
|  | Other | 5 |  |
|  | N/A | 6 |  |
|  | Cannot tell | 99 |  |
| **FOR MESSAGE AS A WHOLE** | | | |
| ***BBJ VALUES*** | | | |
| Safe Space | | | |
|  | Yes | 1 |  |
|  | No | 0 |  |
| Community Service | | | |
|  | Yes | 1 |  |
|  | No | 0 |  |
| Celebration of Black boys and Men | | | |
|  | Yes | 1 |  |
|  | No | 0 |  |
| Protection | | | |
|  | Yes | 1 |  |
|  | No | 0 |  |
| Finding/displaying joy  The ideal is smiling, men looking happy | | | |
|  | Yes | 1 |  |
|  | No | 0 |  |
| ***BMC Values*** | | | |
| Education Black boys and men | | | |
|  | Yes | 1 |  |
|  | No | 0 |  |
| Mentorship | | | |
|  | Yes | 1 |  |
|  | No | 0 |  |
| Attire | | | |
|  | Casual | 1 |  |
|  | Business Casual | 2 |  |
|  | Professional | 3 |  |
|  | Formal | 4 |  |
|  | Stereotypically dressed (i.e. thug garb) | 5 |  |
|  | N/A | 6 |  |
|  | Other | 7 |  |
| If formal, wedding party? | | | |
|  | Yes | 1 |  |
|  | No | 0 |  |
|  | N/A | 2 |  |
| Engaging in atypical gender behaviors  e.g. Incompetent, irresponsible, over-sexed, threat to white notions of manhood, family, and nation | | | |
|  | Yes | 1 |  |
|  | No | 0 |  |
| ***CHALLENGING MASCULINE NORMS^1^*** | | | |
| Restricted affection | | | |
|  | Yes | 1 |  |
|  | No | 0 |  |
| Restricted emotion | | | |
|  | Yes | 1 |  |
|  | No | 0 |  |
| Conflicts  B/w work and family/work and school/school and work | | | |
|  | Yes | 1 |  |
|  | No | 0 |  |
| Competition/Power | | | |
|  | Yes | 1 |  |
|  | No | 0 |  |
|  |  |  |  |
| ***BLACK MASCULINITY^2^*** | | | |
| Cool Pose | | | |
|  | Yes | 1 |  |
|  | No | 0 |  |
| Comedic presence | | | |
|  | Yes | 1 |  |
|  | No | 0 |  |
|  |  |  |  |
| **OTHER CODES (STANDARD)** | | | |
| Message Framing | | | |
|  | Episodic | 1 |  |
|  | Thematic | 2 |  |
|  | Both | 3 |  |
|  | None | 4 |  |
| Likes Frequency |  |  |  |
| Hashtag Frequency |  |  |  |
| List Hashtags |  |  |  |
| Comments Frequency |  |  |  |
| Views Frequency |  |  |  |
| Regram Frequency |  |  |  |
| Repost | | | |
|  | Yes | 1 |  |
|  | No | 0 |  |
| Mentions Frequency |  |  |  |
| List Mentions |  |  |  |
| Hyper link | | | |
|  | Yes | 1 |  |
|  | No | 0 |  |
| If so, what type of link? | | | |
|  | Blog | 1 |  |
|  | Social Media | 2 |  |
|  | Gov’t/regulatory | 3 |  |
|  | Official medical site | 4 |  |
|  | Other health-focused | 5 |  |
|  | Commercial | 6 |  |
|  | Academic | 7 |  |
|  | Other | 8 |  |
|  | None | 9 |  |
|  | Broken link | 10 |  |

**END OF BOOK**

**Operationalizations for Variables**

A couple notes:

**Quote**: will code yes for posts that present an actual quote with quotation marks around it or posts like this:


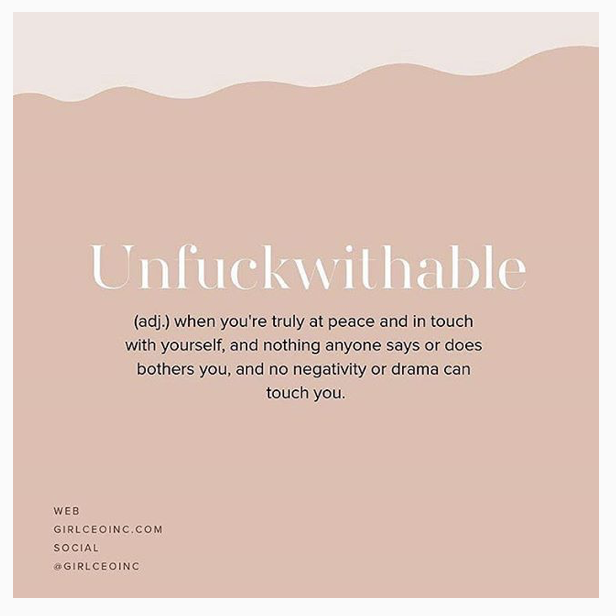

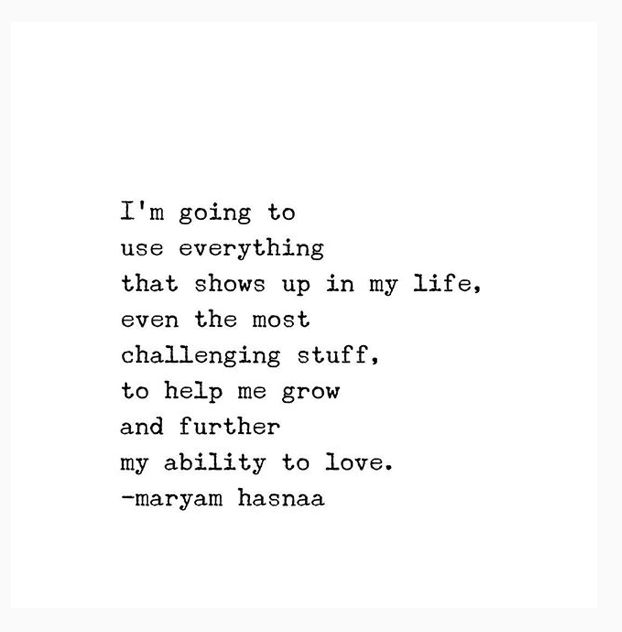


**Type of Quote:** will code as inspirational or motivational, if the quote is positive, encouraging, etc.

*Black Boy Joy Values*

- **Safe Space:** if someone mentions being in a place or going to a place where they can be themselves, free of judgement, be free, etc.
- **Celebrating/Empowering Black boys/men**: if someone, not the poster, posts about a Black men or boy and in the caption or hashtags says something positive about them, e.g. congratulating them, or is shouting them out, thanking them, etc. (note: that the poster can be in the picture with the person they’re shouting out); for instance, when I post about my friends, saying how I appreciate them but I’m also still in the photo with them)
- **Protection**: if a father or man explicitly states something about protecting another boy (maybe son) or man
- **Find Joy/Positive Affect**: ONLY black men/boys who are smiling, look happy and/or mentions in their captions/hashtags about being happy (not killing their vibe?)
- **Education** (general): any mention of education in general (e.g. receiving a degree, attending classes, graduating)
- **Celebrating/Empowering Black boys/men**: if someone, not the poster, posts about a Black men or boy and in the caption or hashtags says something positive about them, e.g. congratulating them, or is shouting them out, thanking them, etc. (note: that the poster can be in the picture with the person they’re shouting out); for instance, when I post about my friends, saying how I appreciate them but I’m also still in the photo with them)
- **If formal, wedding party**: code 2 if not applicable, only code 0 if formally dressed (i.e. gown, tux) but is not part of a wedding party or is not going to a wedding)
- **Engaging in Atypical Gender behaviors**: for black men or boys who engage in behaviors or interests that are typically deemed feminine in today’s society (e.g. playing with dolls, taking dance classes (e.g. ballet); a father doing their daughter’s hair; fathers taking care of child their children to school (could be a single-father); or some might mention taking on “mommy duties”)
- **Restricted Affection**: having limited ways to express one’s feelings and thoughts with other men and difficulty touching other men; *will only code yes if we see restricted affection between men or boys*
- **Restrictive Emotion:** having difficulty and fears about expressing one’s feelings and difficulty finding words to express basic emotions
- **Conflict Between Work and Family**: difficulties balancing work-school and family relations resulting in health problems, overwork, stress, and a lack of leisure and relaxation.
- **Power and Competition**: describes personal attitudes about success pursued through competition and power
- **Cool Pose:** Standing with hands folded in front; leg up on wall, stone-face poses (men don’t smile often, sometimes just pose with serious faces), the look off to the side; light skin squint
- **Comedic presence**: if in the visual or caption, there is mention of a guy being funny, always making me smile with jokes, etc.
